# Mechanistic insights into the size-dependent bioaccumulation and phytotoxicity of polyethylene microplastics in tomato seedlings

**DOI:** 10.3389/fpls.2026.1786469

**Published:** 2026-02-18

**Authors:** Yuanzhou Xu, Xueqing Chen, Youxin Xu, Jie Liu, Wenxia Yang, Wei Wei, Bo Zheng, Chunlei Li

**Affiliations:** 1 Key Laboratory of Facilities Horticulture, Weifang University of Science and Technology, Shouguang, Shandong, China; 2 Weifang Vocational College of Food Science and Technology, Weifang, Shandong, China; 3 Weifang Science and Technology Innovation Promotion Center, Weifang, Shandong, China

**Keywords:** indole-3-acetic acid (IAA), oxidative stress, polyethylene microplastics, size-dependent phytotoxicity, variance partitioning analysis

## Abstract

Microplastic (MP) contamination in agricultural soils poses a growing threat to crop health, yet the size-dependent mechanisms governing their uptake and phytotoxicity remain poorly understood. This study investigated the physiological and biochemical responses of tomato (*Solanum lycopersicum L*.) seedlings exposed to polyethylene (PE) microplastics of four distinct particle sizes at a constant mass concentration (1% w/w): T1 (1–2 mm), T2 (0.2–1 mm), T3 (50–200 μm), and T4 (1–50 μm). Results showed that PE exposure significantly inhibited plant growth in a size-dependent manner. The T4 treatment (1–50 μm) caused the most severe phytotoxicity, reducing shoot fresh weight by 42.3% and total root length by 55.1% compared to the control, indicating that micro-sized particles severely restrict root system expansion. This growth retardation was accompanied by aggravated oxidative stress, evidenced by a 263.4% surge in malondialdehyde (MDA) content (reaching 29.8 nmol/g FW) in the T4 group. To mitigate this stress, the antioxidant defense system was significantly activated, with SOD, POD, and CAT activities increasing by 122.2%, 194.1%, and 177.8%, respectively. The bioaccumulation of PE in plant tissues was highly non-linear and fitted well to the Freundlich isotherm model (R^2^ > 0.97). Notably, the uptake and mobility of MPs were strongly governed by particle size, as reflected by the bioconcentration factor (BCF) and translocation factor (TF). Both indices exhibited a sharp increase as particle size decreased: root BCF surged from 0.016 (T1) to 0.840 (T4), while TF rose from 0.125 to 0.286, confirming the exponentially higher bioavailability and upward translocation potential of micro-sized particles (T4). Physiologically, small-sized MPs (T3, T4) induced non-stomatal limitations to photosynthesis. Furthermore, Variance Partitioning Analysis (VPA) revealed a distinct mechanistic shift around a critical size threshold: while growth inhibition under large-sized MP exposure (T1, T2) was primarily driven by disruptions in indole-3-acetic acid (IAA) homeostasis (explaining 32.4% of variation), the toxicity of small-sized MPs was predominantly governed by oxidative stress responses (explaining 38.6% of variation). These findings highlight that environmental risk assessments based solely on mass concentration may underestimate the hazards of micro-sized fragments, which exert toxicity through fundamentally different physiological pathways compared to larger particles. Ultimately, the identified shifts in hormonal balance and oxidative status provide a valuable mechanistic framework for evaluating the potential impacts of microplastic stress on the nutritional composition and overall quality of tomato fruits in subsequent growth stages.

## 1 Introduction

Polyethylene (PE) mulch films are integral to modern high-intensity agriculture, yet their widespread use has turned agricultural soils into a major sink for plastic residues (Tian et al., 2024). Under continuous weathering forces such as UV radiation and tillage, these residues fragment into microplastics (MPs, < 5 mm) with heterogeneous particle sizes (Cai et al., 2023). As emerging persistent contaminants, MPs not only deteriorate soil structure but also interact with crops, raising profound concerns regarding agro-ecosystem stability and food safety (Ju et al., 2025). While the phytotoxicity of MPs-manifested as growth retardation and oxidative stress-is well-documented, current ecological risk assessments predominantly rely on mass-based concentrations (e.g., mg kg⁻¹). This conventional approach often overlooks a critical determinant of environmental hazard: particle size (Deng et al., 2024).

Recent evidence supports a “size-effect” hypothesis, where phytotoxicity intensifies exponentially as particle size decreases (An et al., 2024; Zhang et al., 2024a). However, the specific uptake kinetics governing this phenomenon remain debated, particularly whether MP bioaccumulation follows non-linear models like the Freundlich isotherm (Cui et al., 2023). More importantly, a fundamental knowledge gap exists regarding the mechanistic shift driving toxicity across different size fractions (Ullah et al., 2025). It remains unclear whether the “size effect” represents merely an intensification of general stress or a distinct transition in the physiological mode of action, specifically shifting from physical hormonal disruption to chemical oxidative damage.

Existing literature suggests a potential dichotomy: macro-sized MPs may primarily act as physical stressors, altering soil aeration and creating mechanical impedance that indirectly downregulates root-regulating hormones like indole-3-acetic acid (IAA) (Xu et al., 2023). In contrast, micro-sized particles (< 50 μm) are hypothesized to breach root barriers, causing intracellular bioaccumulation that triggers severe reactive oxygen species (ROS) bursts (Wang et al., 2021; Zhang et al., 2023). Determining the specific contribution of these divergent pathways-hormonal disruption versus oxidative stress-and identifying the critical size threshold where the dominant driver shifts, is essential for accurate risk stratification (Rawat and Laxmi, 2025; Chen et al., 2024).

To decode these size-dependent mechanisms, this study exposed tomato (*Solanum lycopersicum L*.) seedlings to PE microplastics of four distinct size classes (1–2 mm to < 50 μm) under a constant mass concentration. By integrating physiological assays with variance partitioning analysis (VPA), this research aims to: (1) characterize the non-linear bioaccumulation and translocation kinetics of MPs; (2) elucidate the distinct impacts of particle size on photosynthetic carbon assimilation and root architecture; and (3) quantitatively differentiate the drivers of phytotoxicity, specifically the shift from IAA homeostasis disruption to oxidative stress responses. These findings challenge the sufficiency of mass-based metrics and provide mechanistic insights vital for refining regulatory frameworks for microplastic pollution in agriculture.

## 2 Materials and methods

### 2.1 Soil sampling and characterization

Test soil was collected from the surface layer (0–20 cm) of an agricultural field in Nanjing, Jiangsu Province, China, during the crop fallow period in June 2025. This site had no history of plastic film mulching. The collected soil was air-dried, ground, and sieved through a 2-mm mesh to remove coarse debris. The physicochemical properties of the soil were determined as follows: pH 6.8, organic matter content 15.2 g kg⁻¹, and a sandy loam texture comprising 65.3% sand, 22.1% silt, and 12.6% clay.

### 2.2 Preparation and fractionation of microplastics

To simulate a realistic contamination scenario, waste polyethylene (PE) agricultural mulch films collected from local farmlands were used as the microplastic source. The films were first rinsed with tap water, followed by ultrasonic cleaning in anhydrous ethanol and deionized water for 30 min each to remove soil particles and potential agrochemical residues.

The cleaned films were air-dried, embrittled using liquid nitrogen, and pulverized using a cryogenic grinder. The resulting powder was fractionated into four distinct size classes using standard stainless-steel sieves via a wet-sieving method (95% ethanol). The fractions were defined as follows: 1–2 mm (T1), 0.2–1 mm (T2), 50–200 μm (T3), and < 50 μm (T4). The T4 fraction was obtained by centrifuging the filtrate that passed through the 50-μm sieve. All microplastic particles were dried at 50 °C and sterilized under UV light for 12 h prior to application.

### 2.3 Plant material and experimental design

Tomato seeds (Solanum lycopersicum L., cv. Zhongshu No. 4) were surface-sterilized with 5% sodium hypochlorite (NaClO) for 10 min and rinsed thoroughly with deionized water. Seeds were germinated on moist filter paper in the dark, and uniform seedlings with 3–4 true leaves were selected for transplantation.

A pot experiment was conducted in a climate-controlled greenhouse (25/18 °C day/night temperature, 16 h photoperiod). A randomized complete block design was employed with five treatments; each replicated four times: CK: Soil without microplastic addition (Control); T1: Soil spiked with 1–2 mm PE particles; T2: Soil spiked with 0.2–1 mm PE particles; T3: Soil spiked with 50–200 μm PE particles; T4: Soil spiked with < 50 μm PE particles.

PE particles were added to the soil at a concentration of 1% (w/w) (10 g kg⁻¹). To ensure homogeneity, a stepwise mixing procedure was used: 10 g of PE was first premixed with 100 g of soil, which was then thoroughly mixed with the remaining 890 g of soil. To validate the uniformity of PE distribution, soil samples were randomly collected from different depths of the pots prior to transplantation. The actual PE concentrations were quantified using the FTIR-ATR method described below (Section 2.7), confirming that the variation coefficient was less than 5%, indicating satisfactory homogeneity. Each pot contained 1 kg of soil and one tomato seedling. Soil moisture was maintained at 60% of field water holding capacity by daily gravimetric weighing (weighing pots daily and adding deionized water to reach the target weight) to minimize water potential variations caused by MP addition. The exposure period lasted for 45 days.

### 2.4 Determination of plant growth and root morphology

At harvest, plants were separated into shoots and roots. Roots were gently rinsed under running tap water to remove bulk soil, followed by three rinses in deionized water. Before scanning, roots were floated in water and cleaned using fine tweezers to remove any remaining particles to ensure high contrast for image analysis. Fresh weights (FW) were recorded immediately. Root morphological traits (total length and surface area) were analyzed using a root scanner (Epson Expression 12000XL) coupled with WinRHIZO software (Regent Instruments Inc., Canada).

### 2.5 Measurement of photosynthetic parameters

In accordance with the method of Xu et al. (2023), Gas exchange parameters, including net photosynthetic rate (Pn), transpiration rate (Tr), stomatal conductance (Gs), and intercellular CO_2_ concentration (Ci), were measured on the third fully expanded leaf between 9:00 and 11:00 AM using a portable photosynthesis system (Li-Cor 6800).

### 2.6 Quantification of endogenous IAA content

Endogenous IAA was extracted and quantified following a modified protocol based on Xu et al. (2025). Briefly, fresh tissue samples (0.1 g) were ground in liquid nitrogen and extracted with 2 mL of cold 80% methanol (4 °C, 12 h). After centrifugation (12,000 rpm, 5 min, 4 °C), the supernatant was purified using a preconditioned C18 solid-phase extraction cartridge. Impurities were removed with 20% methanol containing 0.1% formic acid, and the target IAA fraction was retrieved using 1 mL of 80% methanol.

Chromatographic separation was performed on a Finnigan LC–MS/MS system (Thermo Electron, USA) equipped with a HiQ Sil C18 column (250 mm × 4.6 mm, 5 μm). An isocratic elution was applied using a mixture of methanol and water (80:20, v/v) containing 0.1% formic acid at a flow rate of 1.0 mL min⁻¹. The injection volume was 25 μL. Mass spectrometric detection was conducted using an electrospray ionization (ESI) source in positive ion mode. Key operating parameters were set as follows: spray voltage, 4 kV; capillary temperature, 350 °C; and capillary voltage, 70 V. Quantification was achieved in selected reaction monitoring (SRM) mode by monitoring the transition m/z 174 → 130, with a collision energy of 15 eV. The recovery rate and detection limit of the method were validated prior to analysis.

### 2.7 Microplastic uptake and tissue accumulation

Microplastic was extracted and quantified following a modified protocol based on Rathore et al. (2023). The uptake and accumulation of PE microplastics in plant tissues were identified and quantified using Fourier Transform Infrared Spectroscopy equipped with an Attenuated Total Reflection accessory (FTIR-ATR). Prior to analysis, root and leaf samples were thoroughly washed, freeze-dried, and ground into a fine powder. To minimize experimental errors arising from heterogeneous distribution, the powdered samples were mixed thoroughly to ensure homogeneity before spectral acquisition.

Spectral measurements were performed in the range of 4000–400 cm⁻¹ using an FTIR spectrometer (Thermo Nicolet iS50). The characteristic absorption peak at 2850 cm⁻¹, corresponding to the symmetric stretching vibration of the methylene group (-CH_2_-), was selected as the diagnostic marker for PE identification.

For quantitative analysis, the integrated peak area at 2850 cm⁻¹ was used as the quantitative index. A standard calibration curve was established by plotting the peak areas against a series of known PE concentrations mixed with the plant matrix. The method showed high linearity (R^2^≥0.99). The PE concentration in the tissue samples (mg kg⁻¹) was calculated by substituting the integrated peak areas of the samples into the established regression equation.

The Bioconcentration Factor (BCF) and Translocation Factor (TF) were calculated as follows: BCF=C_tissue_/C_soil_; TF= C_leaf_/_Croot_.

Where C_tissue_, C_soil_, C_leaf_, and C_root_ represent the PE concentrations in the plant tissue, soil, leaves, and roots, respectively.

### 2.8 Analysis of oxidative stress and antioxidant enzymes

Following the method of Xu et al. (2023), lipid peroxidation was assessed by measuring malondialdehyde (MDA) content via the thiobarbituric acid (TBA) assay. The activities of superoxide dismutase (SOD), peroxidase (POD), and catalase (CAT) were determined spectrophotometrically at 560, 470, and 240 nm, respectively.

### 2.9 Statistical analysis

Data are expressed as mean ± standard deviation (SD) (n=4). Statistical differences were analyzed using one-way ANOVA followed by Duncan’s multiple range test (p < 0.05) using SPSS 26.0. Principal Component Analysis (PCA) was conducted to visualize the overall physiological responses. The relationship between particle size and tissue accumulation was fitted using the Freundlich isotherm model:


$$q_{e}=\;=K_{F}\cdot Ce^{\frac{1}{n}}$$

Where qe is the PE concentration in tissues, Ce represents the exposure availability (related to specific surface area or inverse of particle size), and the parameters KF and n represent the adsorption capacity and surface heterogeneity, respectively, as applied in plant uptake studies (Li et al., 2005; Liu et al., 2021). Variance Partitioning Analysis (VPA) was performed using the vegan package in R software (version 4.2.3) to quantify the relative contribution of IAA content and antioxidant enzyme activities to the observed variations in PE toxicity across different particle size groups.

## 3 Results

### 3.1 Impact of PE particle size on tomato growth and root development

**Figure 1:**
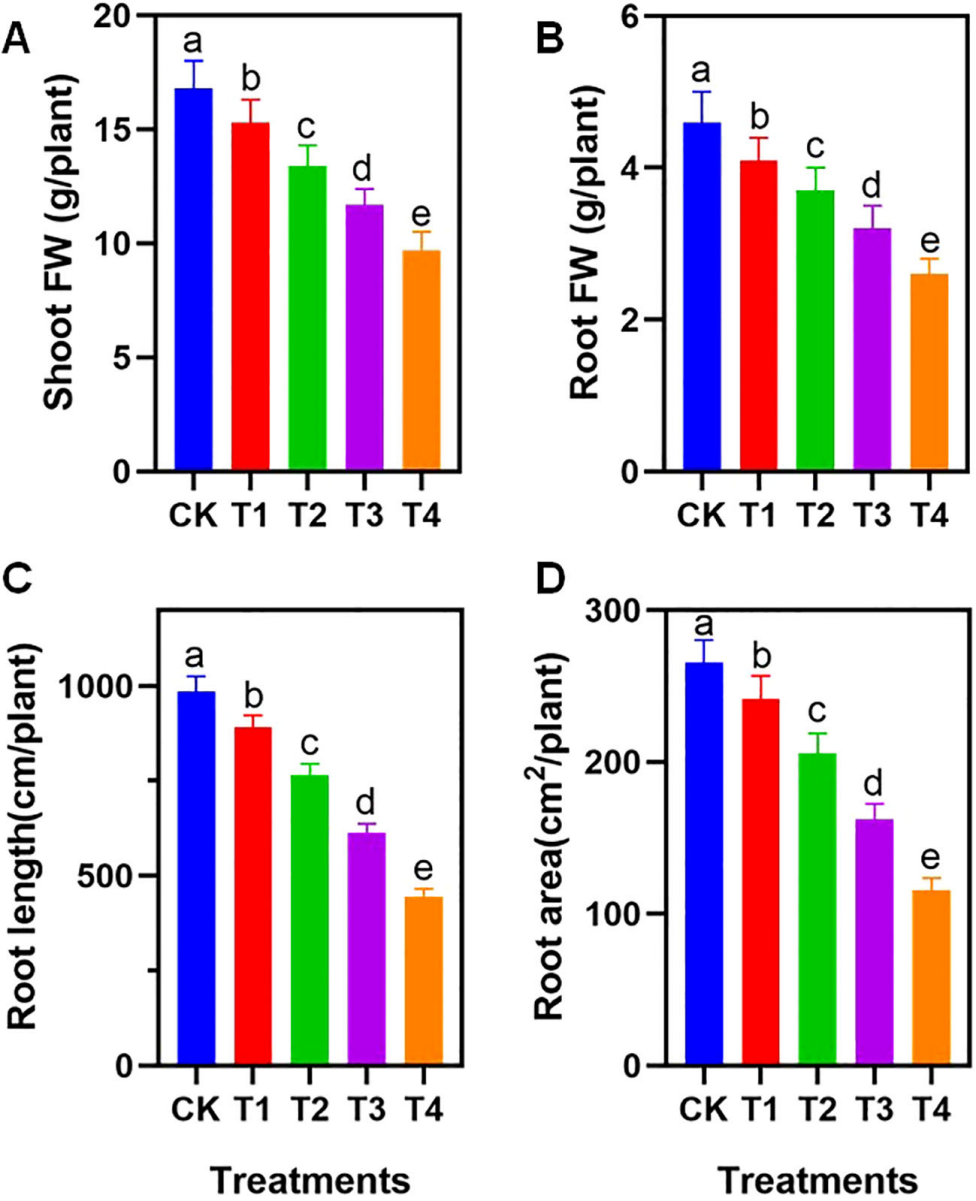
Effects of different PE particle sizes on tomato growth and root morphological characteristics. **(A)** Aboveground and **(B)** underground fresh weight of tomato; **(C)** Total root length of tomato; **(D)** Root surface area of tomato. CK: no PE, T1: 1-2mm, T2: 0.2-1mm, T3: 50-200μm, T4: 1-50μm. Different letters on the tops of columns indicate significant differences at p < 0.05. Error bars represent standard deviation.

Exposure to polyethylene (PE) microplastics resulted in a significant, particle size-dependent inhibition of tomato seedling growth. Phenotypic measurements showed a progressive decline in biomass and root development as particle size decreased from 1–2 mm (T1) to 1–50 μm (T4) (**Figure 1**).

Compared to the control (CK), PE treatments significantly reduced both shoot and root fresh weights (FW) (p < 0.05). The T4 treatment exhibited the lowest biomass, with reductions of 42.3% in shoots and 43.1% in roots relative to the CK (**Figures 1A, B**). Root morphological traits followed a similar downward trend. Total root length and root surface area in the T4 group were reduced by 55.1% and 56.4%, respectively, compared to the CK (**Figures 1C, D**), confirming that the inhibitory effect on root expansion was most pronounced in the smallest particle size treatment.

### 3.2 Response of photosynthetic characteristics

PE exposure significantly altered the photosynthetic parameters of tomato seedlings, with distinct response patterns observed across different particle sizes (**Table 1**). Net photosynthetic rate (Pn), transpiration rate (Tr), and stomatal conductance (Gs) decreased progressively as particle size diminished (p < 0.05). The T4 treatment showed the lowest values, with Pn and Gs reduced by 52.8% and 68.8% respectively, compared to the CK.

**Table 1:** Net photosynthetic rate (Pn), transpiration rate (Tr), intercellular CO_2_ concentration (Ci), and stomatal conductance (Gs) in control and PE-treated tomato seedlings under different particle size treatments.

Treatments	Pn (μmol m^-2^s^-1^)	Tr (μmol m^-2^s^-1^)	Ci (μmol mol^-1^)	Gs (μmol m^-2^s^-1^)
CK	18.0 ± 1.2	4.8 ± 0.3	285 ± 10	0.32 ± 0.02
T1	16.5 ± 1.0	4.2 ± 0.2	270 ± 8	0.28 ± 0.02
T2	13.8 ± 0.9	3.5 ± 0.2	255 ± 9	0.22 ± 0.01
T3	10.2 ± 0.8	2.7 ± 0.2	290 ± 11	0.15 ± 0.01
T4	8.5 ± 0.7	2.1 ± 0.1	320 ± 12	0.10 ± 0.01

Data are means ± standard error of four replicates (n = 4). Values followed by different letters within same column are significantly different at p < 0.05 significance level according to the Duncan’s multiple range test. CK: no PE, T1: 1-2mm, T2: 0.2-1mm, T3: 50-200μm, T4: 1-50μm.

Notably, the intercellular CO_2_ concentration (Ci) exhibited a non-monotonic response. While Ci decreased slightly in the T1 and T2 treatments, it significantly increased in the T3 (290 μmol mol⁻¹) and T4 (320 μmol mol⁻¹) groups (p < 0.05). The observation of decreased Pn accompanied by increased Ci in the T3 and T4 treatments indicates that the photosynthetic inhibition in these groups was primarily attributed to non-stomatal limitations rather than stomatal closure.

### 3.3 Changes in endogenous IAA concentrations

**Figure 2:**
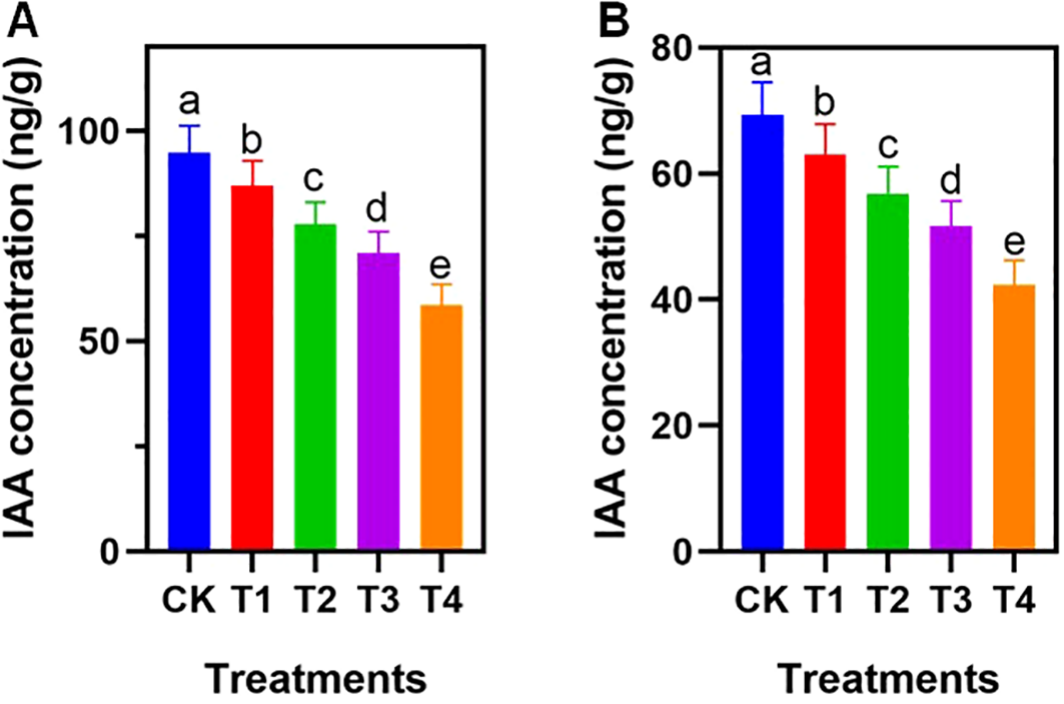
Effect of different PE particle sizes on IAA concentrations. Data on **(A)** leaf IAA and **(B)** root IAA are presented. CK: no PE, T1: 1-2mm, T2: 0.2-1mm, T3: 50-200μm, T4: 1-50μm. Different letters on the tops of columns indicate significant differences at P < 0.05. Error bars represent standard deviation.

The concentration of indole-3-acetic acid (IAA) in both leaves and roots was significantly affected by PE exposure (**Figure 2**). A consistent size-dependent decline was observed across tissues. In leaves, IAA levels dropped progressively, with the T4 treatment showing a 38.2% reduction compared to the CK (**Figure 2A**).

In roots, the decrease was similarly pronounced. Root IAA concentrations fell from 69.5 ng/g in the CK to 42.3 ng/g in the T4 treatment, representing a 39.1% reduction (**Figure 2B**). Statistical analysis confirmed significant differences among treatments (p < 0.05), indicating that the presence of smaller PE microplastics corresponded to lower levels of endogenous auxin in the plant tissues.

### 3.4 Uptake, bioaccumulation, and translocation of PE microplastics

PE microplastics were detected in the root and leaf tissues of all treated plants, with uptake and distribution patterns varying significantly by particle size (**Table 2**). No PE was detected in the control group.

**Table 2:** PE concentrations in the leaves and roots of tomato seedlings, bioconcentration factors (BCF), and translocation factor (TF).

PE treatment	Leaf PE conc (mg/kg)	Root PE conc (mg/kg)	Leaf BCF	Root BCF	TF
CK	ND	ND	ND	ND	ND
T1	0.01 ± 0.002d	0.08 ± 0.009d	0.002 ± 0.0001d	0.016 ± 0.001d	0.125 ± 0.01d
T2	0.12 ± 0.01c	0.45 ± 0.03c	0.024 ± 0.002c	0.090 ± 0.008c	0.267 ± 0.02c
T3	0.45 ± 0.02b	1.80 ± 0.12b	0.090 ± 0.008b	0.360 ± 0.03b	0.250 ± 0.02b
T4	1.20 ± 0.05a	4.20 ± 0.21a	0.240 ± 0.02a	0.840 ± 0.06a	0.286 ± 0.02a

Data are means ± standard error of four replicates (n = 4). Values followed by different letters within same column are significantly different at p < 0.05 significance level according to the Duncan’s multiple range test. CK: no PE, T1: 1-2mm, T2: 0.2-1mm, T3: 50-200μm, T4: 1-50μm.

The concentration of PE in tissues increased as particle size decreased (p < 0.05). In the T4 treatment, PE concentrations reached 4.20 mg/kg in roots and 1.20 mg/kg in leaves, which were significantly higher than those in the T1 treatment. Consequently, the translocation factor (TF) increased from 0.125 in T1 to 0.286 in T4. Although all TF values were below 1.0, the higher TF in the T4 group indicates a relatively greater distribution of particles to the aerial parts in the 1–50 μm treatment compared to the larger size treatments.

### 3.5 Oxidative stress and antioxidant enzyme activities

**Figure 3:**
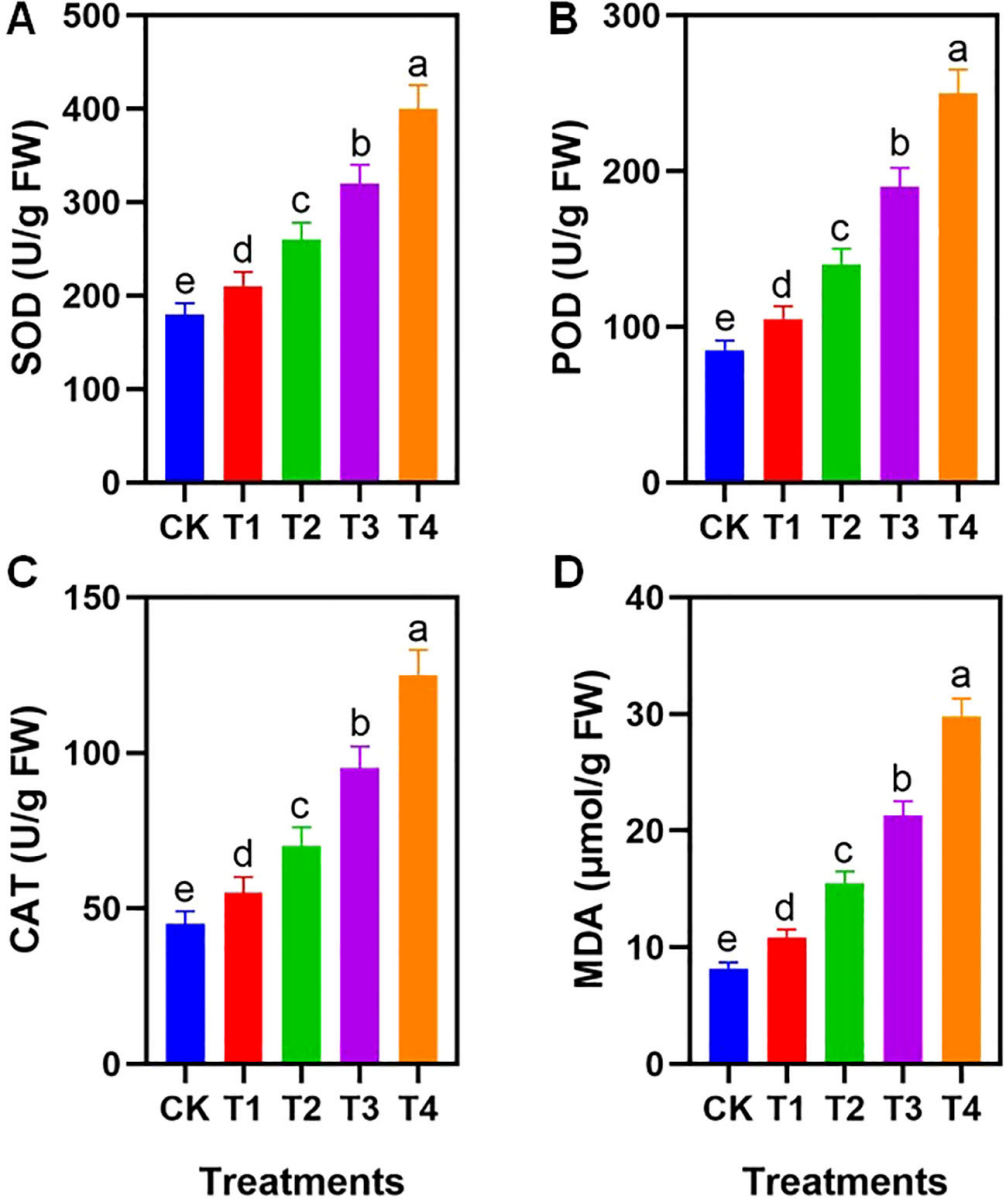
Effects of different PE particle sizes on antioxidant enzyme activity. Data on **(A)** Superoxide dismutase (SOD), **(B)** Peroxidase (POD), **(C)** Catalase (CAT), and **(D)** Malondialdehyde (MDA) contents are presented. CK: no PE, T1: 1-2mm, T2: 0.2-1mm, T3: 50-200μm, T4: 1-50μm. Different letters on the tops of columns indicate significant differences at P < 0.05. Error bars represent standard deviation. FW indicates fresh weight.

PE exposure induced significant changes in oxidative stress markers and antioxidant enzyme activities (**Figure 3**). The activities of superoxide dismutase (SOD), peroxidase (POD), and catalase (CAT) increased significantly as the PE particle size decreased (p < 0.05). The highest enzymatic activities were recorded in the T4 treatment, where SOD, POD, and CAT levels exceeded the CK by 1.23-fold, 1.94-fold, and 1.79-fold, respectively.

Concurrently, the content of malondialdehyde (MDA), a marker of lipid peroxidation, rose significantly in the smaller particle treatments. The MDA content in the T4 group was 3.67 times higher than that of the CK (**Figure 3D**). Despite the significant upregulation of SOD and CAT activities, the MDA content in the T4 treatment surged by 263.4%, indicating that the rate of ROS generation induced by micro-sized particles exceeded the scavenging capacity of the activated antioxidant system.

### 3.6 Multivariate statistical analysis of toxicity drivers

**Figure 4:**
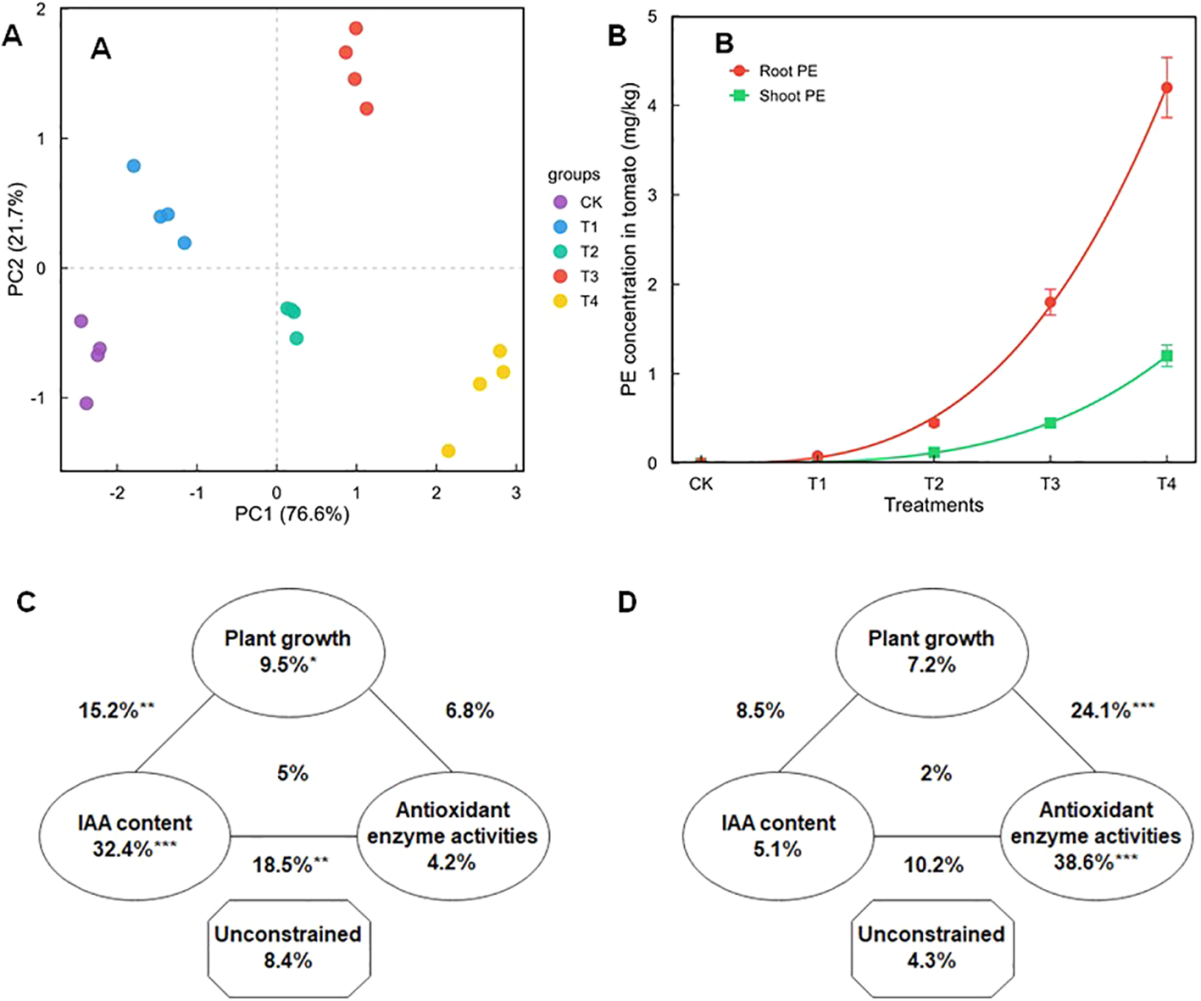
**(A)** Principal component analysis (PCA) score plot illustrating the variance between the effects of different PE particle sizes. **(B)** PE contents in tomato seedlings under different treatments. Solid lines represent data that fit the Freundlich model. Variance partitioning analysis (VPA) map of the effects of plant growth, IAA concentration, antioxidant enzyme activities, and the interactions of these factors on PE uptake under **(C)** large-sized and **(D)** small-sized PE treatments. CK: no PE, T1: 1-2mm, T2: 0.2-1mm, T3: 50-200μm, T4: 1-50μm. Numbers represent the contribution of each environmental factor alone and the interaction of different environmental factors to PE uptake. One, two, and three asterisks represent significance at P < 0.05, P < 0.01, and P < 0.001, respectively.

Principal component analysis (PCA) revealed a distinct separation of treatment groups, with PC1 accounting for 76.6% of the total variance, illustrating a clear trajectory of physiological changes associated with decreasing particle size (**Figure 4A**). The PCA plot revealed distinct clustering: the Control, T1, and T2 treatments clustered closely, suggesting similar physiological states dominated by physical stress. In contrast, T3 and T4 formed a separate cluster along the PC1 axis, indicating a divergent physiological response characterized by oxidative stress.

The accumulation data for both roots and leaves fitted well to the Freundlich isotherm model (R^2^ > 0.97), describing the non-linear relationship between particle size and tissue concentration (**Figure 4B**). Furthermore, Variance Partitioning Analysis (VPA) was used to quantify the relative contribution of different physiological factors to the observed variance (**Figures 4C, D**). In the large particle treatments (T1, T2), IAA content explained the largest proportion of variance (32.4%, p < 0.001). In contrast, in the small particle treatments (T3, T4), antioxidant enzyme activity became the dominant explanatory factor (38.6%, p < 0.001). This shift in explanatory variables indicates distinct physiological response patterns between the large-sized (T1, T2) and small-sized (T3, T4) PE treatments.

Correlation analysis demonstrated that BCF (including Root.BCF and Leaf.BCF) was positively correlated with PE accumulation (Root.PE and Leaf.PE), antioxidant enzyme activities (SOD, CAT, and POD), MDA content, TF, and Ci, but negatively correlated with biomass (Shoot.FW and Root.FW), root morphology (Root.length and Root.area), IAA content (Leaf.IAA and Root.IAA), and photosynthetic parameters (Pn, Tr, and Gs). IAA content was found to be positively correlated with biomass, root morphology, and gas exchange parameters (Pn, Tr, and Gs) and negatively correlated with PE accumulation, BCF, antioxidant activities, and MDA content. Furthermore, TF was positively correlated with Leaf.BCF and Leaf.PE, and Pn was positively correlated with Tr and Gs (**Supplementary Figure S1**).

## 4 Discussion

### 4.1 Size-dependent responses of tomato growth and physiological indices to PE microplastics

#### 4.1.1 Plant biomass and root morphological traits

Exposure to PE microplastics (MPs) resulted in a pronounced, size-dependent inhibition of tomato seedling growth, validating the “size-effect” hypothesis observed in recent studies where phytotoxicity intensifies as particle size decreases (An et al., 2024; Zhang et al., 2024a). The T4 treatment (1–50 μm) caused the most severe biomass reduction (**Figures 1A, B**), suggesting that micro-sized particles exert a higher stress burden than macro-sized fragments despite identical mass concentrations (1% w/w). This inhibition was most evident in the root system, where the significant reduction in total root length and surface area (**Figures 1C, D**) indicates that smaller MPs may physically block root pores or adhere to the rhizodermis, creating a mechanical barrier that impedes water and nutrient acquisition (Wong and Taylor, 2025). The compromised root architecture in the T4 group severely limited the absorptive surface area, creating a nutritional deficit that subsequently suppressed shoot expansion (Lopez et al., 2023). The positive correlation between root morphological structure and biomass was confirmed in this study (**Supplementary Figure S1**).

#### 4.1.2 Photosynthetic and transpiration parameters

The inhibition of plant growth was physiologically underpinned by compromised photosynthetic performance. Crucially, our data revealed distinct mechanisms of photosynthetic limitation driven by particle size. In the T1 and T2 treatments (large particles), the parallel decline in net photosynthetic rate (Pn) and intercellular CO_2_ (Ci) points to stomatal limitation (Warren, 2004). In the large-sized MP treatments (T1, T2), a significant positive correlation was observed between stomatal conductance (Gs) and Pn (p < 0.05). This strong correlation confirms that the reduction in carbon assimilation was primarily constrained by stomatal closure. This suggests that large MPs likely altered soil physical properties, triggering a water-deficit signal that prompted stomatal closure. Conversely, in the T3 and T4 treatments (small particles), Pn decreased while Ci significantly increased. This “high Ci, low Pn” pattern is a hallmark of non-stomatal limitation (Ma et al., 2025). The accumulation of intercellular CO_2_ indicates that mesophyll cells lost the capacity to fix carbon efficiently, likely due to damage to the photosynthetic apparatus (e.g., thylakoid membranes) or inhibition of Calvin cycle enzymes (Li et al., 2023).

#### 4.1.3 Endogenous IAA content

Root system architecture is strictly regulated by phytohormones, particularly IAA, which governs cell elongation and meristematic activity. The significant, size-dependent reduction in IAA content (**Figure 2**) provides a biochemical explanation for the observed root stunting. The depletion of auxin in the T4 treatment suggests that micro-sized MPs may trigger stress signaling pathways that either downregulate auxin biosynthesis genes or accelerate auxin degradation (Xu et al., 2025). This hypothesis is supported by the significant positive correlation between root IAA content and total root length observed in our study (**Supplementary Figure S1**). Since auxin is essential for lateral root emergence, its deficiency directly restricted the expansion of the root system, thereby amplifying the physical toxicity of MPs (Rawat and Laxmi, 2025).

#### 4.1.4 Oxidative stress and antioxidant enzyme activities

The dramatic surge in MDA content in the T3 and T4 treatments (**Figure 3D**) confirms that small-sized MPs induced severe lipid peroxidation, compromising cell membrane integrity. Although the antioxidant defense system was significantly activated-evidenced by upregulated SOD, POD, and CAT activities-the persistence of high MDA levels indicates an insufficient antioxidant response. The rate of ROS generation induced by micro-sized particles clearly exceeded the scavenging capacity of the enzymes (Zhang et al., 2023). Notably, a significant positive correlation was found between MDA content and intercellular CO_2_ concentration (Ci) (**Supplementary Figure S1**), suggesting that oxidative damage to the photosynthetic apparatus was the primary driver of the observed non-stomatal inhibition.

### 4.2 Characteristics and influencing factors of PE microplastic bioaccumulation and translocation in tomato

#### 4.2.1 Relationship between plant growth, root morphology and PE bioaccumulation

The bioaccumulation of PE MPs followed a non-linear trend fitted to the Freundlich isotherm model (**Figure 4B**), indicating that uptake is governed by surface adsorption kinetics. The exponential increase in root bioconcentration factor (BCF) in the T4 group coincided with the reduced root surface area. Correlation analysis revealed a negative relationship between root BCF and root morphological traits (length and surface area) (**Supplementary Figure S1**). This suggests a feedback mechanism: while smaller roots have less total surface area, the specific sites available for adsorption (e.g., root tips, lateral root cracks) become saturated with small MPs due to their high specific surface energy, leading to intense localized toxicity that further inhibits growth (Miao et al., 2019).

#### 4.2.2 Relationship between endogenous IAA content and PE bioaccumulation

A strong negative correlation was observed between IAA content and PE accumulation (**Supplementary Figure S1**). This implies that high tissue concentrations of MPs may disrupt polar auxin transport (Zhang et al., 2024b). The accumulation of MPs in vascular tissues could physically impede the acropetal or basipetal transport of hormones, or the induced ROS could oxidatively degrade auxin molecules. High levels of ROS, particularly H2O2, can induce the decarboxylation of the IAA side chain, thereby directly reducing the pool of active auxin available for root elongation (Cheng et al., 2024; Vanneste et al., 2025). Consequently, plants with higher MP loads (T4) exhibited the lowest IAA levels, exacerbating growth inhibition.

#### 4.2.3 Relationship between oxidative stress responses and PE bioaccumulation

The accumulation of PE particles in plant tissues served as a direct trigger for oxidative burst. The positive correlation between BCF and antioxidant enzyme activities (**Supplementary Figure S1**) confirms that internalized MPs act as intracellular stressors. Upon entering the cells or apoplastic space, micro-sized MPs likely interact with organelles (e.g., mitochondria, chloroplasts), disrupting electron transport chains and leading to electron leakage that forms superoxide radicals, thus necessitating the upregulation of SOD and CAT (Xu et al., 2023).

#### 4.2.4 Correlation between photosynthetic parameters and PE translocation

The TF increased significantly in the T4 treatment (**Table 2**), indicating that smaller particles are more mobile within the transpiration stream. The detection of PE in leaves implies transport via the xylem (Guo et al., 2023). The presence of these foreign particles in leaf tissues likely disrupted stomatal regulation and mesophyll conductance, as evidenced by the negative correlation between Leaf BCF and photosynthetic parameters (Pn, Gs) (**Supplementary Figure S1**). This suggests that MP translocation not only poses a food safety risk but directly impairs the plant’s carbon assimilation source (Lu et al., 2025).

### 4.3 Size-dependent phytotoxicity mechanisms of PE microplastics in tomato

#### 4.3.1 Mechanistic differentiation between large-sized and small-sized PE microplastics

A critical finding of this study, elucidated by variance partitioning analysis (VPA), is the distinct mechanistic shift in toxicity drivers based on particle size (**Figures 4C, D**).

Large-sized MPs (T1, T2): Toxicity was primarily driven by disruptions in IAA homeostasis (explaining 32.4% of variation). This suggests that macro-plastics act mainly as physical soil stressors (Xu et al., 2023). They alter soil structure and mechanical impedance, which indirectly downregulates auxin signaling without causing severe intracellular damage (Kumari et al., 2021).

Small-sized MPs (T3, T4): Toxicity was predominantly governed by oxidative stress responses (explaining 38.6% of variation). This indicates a shift to cellular toxicity. Due to their ability to be internalized and interact with cellular components, micro-sized particles trigger immediate biochemical chaos (ROS burst), making oxidative stress the lethal factor (Wang et al., 2021).

#### 4.3.2 Synergistic effects of IAA homeostasis disruption and oxidative stress on PE-induced phytotoxicity

While distinct drivers exist, the toxicity in the smallest size fraction (T4) likely results from the synergistic interplay of both mechanisms. The “physical” blockage of root pores by MPs restricts water uptake, inducing osmotic stress that lowers IAA levels (Jia et al., 2023). Furthermore, the physical blockage of root pores by accumulated MPs may restrict the uptake of essential macronutrients, such as nitrogen and phosphorus, thereby indirectly contributing to the biomass reduction (Azeem et al., 2021; Hua et al., 2024). Simultaneously, the “chemical” stress from internalized MPs generates ROS, which further degrades IAA (Xu et al., 2023). This dual attack-hormonal suppression preventing recovery and oxidative damage destroying cellular machinery-explains why the T4 treatment resulted in the most severe phytotoxicity.

### 4.4 Environmental implications and agricultural risk management

Our findings challenge the sufficiency of current ecological risk assessments, which predominantly rely on mass-based concentrations (e.g., mg kg⁻¹) to define pollution thresholds. While the mass concentration in this study was constant (1% w/w) across all treatments, the ecological hazard varied dramatically, with the smallest particles (T4) causing exponentially higher phytotoxicity and bioaccumulation (Yang et al., 2024). This discrepancy underscores that mass concentration alone underestimates the risks posed by micro-sized fragments. Consequently, future regulatory frameworks and environmental monitoring standards must incorporate particle size distribution and particle number concentration as critical parameters to accurately predict the toxicity of microplastic pollution (Yu and Hu, 2022).

Furthermore, these results provide practical insights for plastic management in agro-ecosystems. The transition from macro- (T1, T2) to micro-sized (T3, T4) particles represents a critical threshold where the mode of toxicity shifts from physical soil alteration to severe cellular oxidative stress and hormonal disruption. This highlights the urgent necessity of removing agricultural mulch films before they degrade into the highly toxic < 50 μm size range (Sander, 2019). Effective management strategies should prioritize the retrieval of macro-plastic residues to prevent the formation of these vectors for contaminant transfer, thereby safeguarding crop productivity and minimizing the risk of trophic transfer to the human food chain (Macheca et al., 2024).

## 5 Conclusions

This study demonstrates that the phytotoxicity and bioaccumulation of PE microplastics (MPs) in tomato seedlings are profoundly governed by particle size, revealing a distinct mechanistic shift around a critical size threshold. Our findings lead to the following key conclusions. The uptake of PE particles into plant tissues follows a non-linear trend well-described by the Freundlich isotherm model (R^2^>0.97). As particle size decreases from 2 mm to < 50 μm, the BCF and TF increase significantly, confirming that micro-sized particles possess exponentially higher bioavailability and upward mobility via the transpiration stream. Furthermore, A fundamental shift in the “Mode of Action” was identified. Large-sized MPs (T1, T2) primarily act as physical soil stressors, inducing stomatal limitations and disrupting IAA homeostasis (explaining 32.4% of variation). In contrast, small-sized MPs (T3, T4) exert severe cellular toxicity through oxidative bursts and non-stomatal photosynthetic inhibition (explaining 38.6% of variation). The T4 treatment (< 50 μm) represents the most hazardous fraction, causing a synergistic “dual attack” of hormonal suppression and irreversible oxidative damage to the photosynthetic apparatus. Moreover, our results challenge the validity of current mass-based ecological risk assessments. While the mass concentration remained constant (1% w/w), the ecological hazard varied dramatically across size fractions. This highlights that relying solely on mass concentration leads to a significant underestimation of the risks posed by micro-sized fragments in agricultural soils. These findings underscore the urgent necessity of prioritizing the retrieval of macro-plastic residues (e.g., mulch films) before they degrade into the highly toxic < 50 μm range. Future regulatory frameworks and monitoring standards must incorporate particle size distribution and particle number concentration as critical metrics to accurately predict the toxicity and food chain risks of microplastic pollution in agro-ecosystems. Future studies should investigate the potential impacts of microplastic stress on the nutritional composition of tomato fruits and the long-term interactions with the rhizosphere microbiome.

## Data Availability

The original contributions presented in the study are included in the article/**Supplementary Material**. Further inquiries can be directed to the corresponding authors.

## Supplementary material

Supplementary Figure 1 Correlation coefficient plot between various indicators. Shoot.FW: Shoot fresh weight; Root.FW: Root fresh weight; Root.length: Root length; Root.area: Root surface area; Leaf.IAA: IAA content in leaf; Root.IAA: IAA content in root; Pn: Net photosynthetic rate; Tr: Transpiration rate; Gs: Stomatal conductance; Ci: Intercellular CO_2_ concentration; Leaf.PE: PE content in leaf; Root.PE: PE content in root; Leaf.BCF: Bioconcentration factor of PE in leaf; Root.BCF: Bioconcentration factor of PE in root; TF: Translocation factor; SOD: Superoxide dismutase; CAT: Catalase; POD: Peroxidase; MDA: Malondialdehyde content.
