# Supplementary figures and images for: Mechanistic insights into the size-dependent bioaccumulation and phytotoxicity of polyethylene microplastics in tomato seedlings
Source: Front Plant Sci. 2026 Feb 18;17:1786469. doi: 10.3389/fpls.2026.1786469 (PMC12957103; doi:10.3389/fpls.2026.1786469)

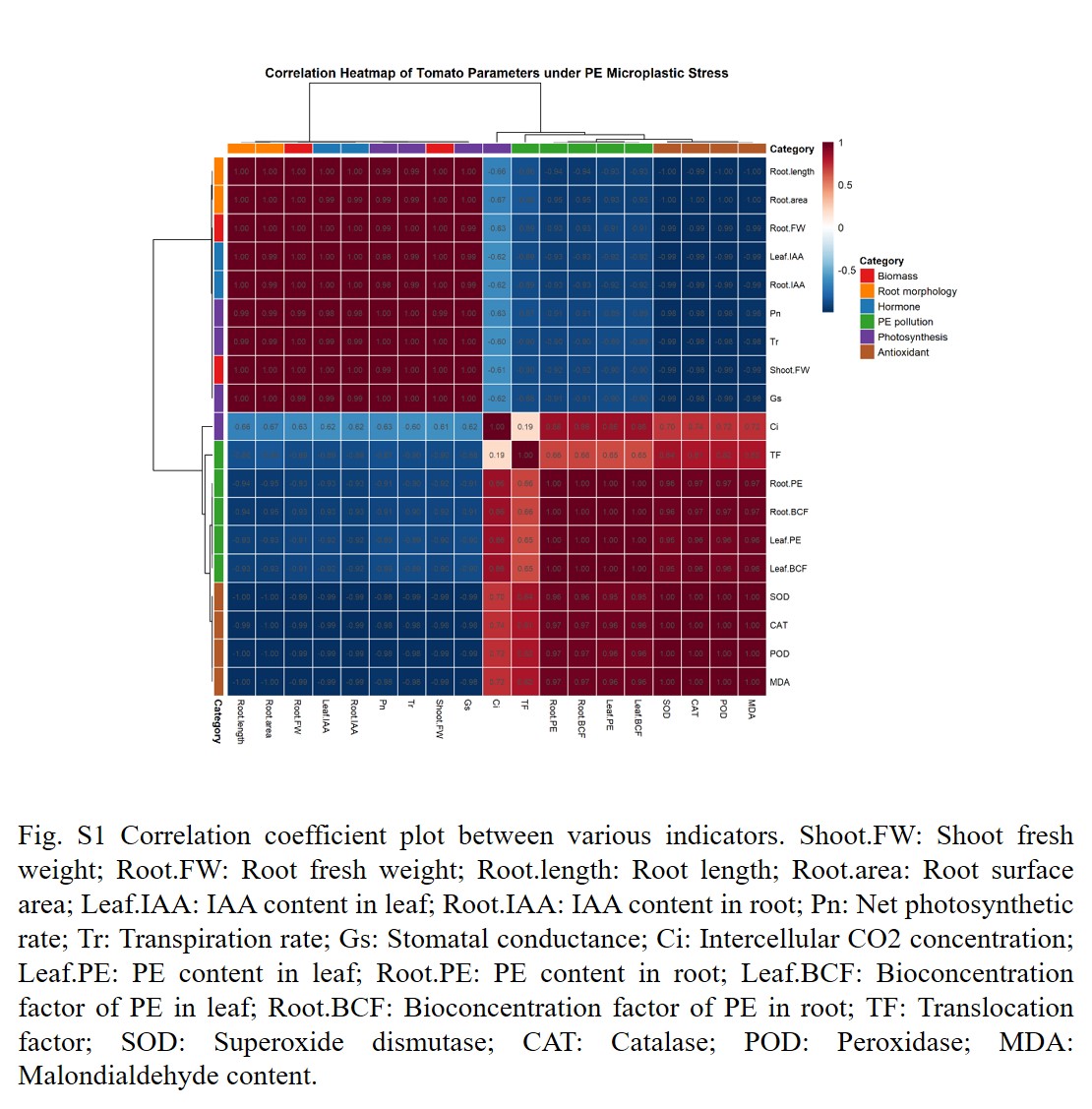

Supplement: Supplementary Figure 1 — Correlation coefficient plot between various indicators. Shoot.FW: Shoot fresh weight; Root.FW: Root fresh weight; Root.length: Root length; Root.area: Root surface area; Leaf.IAA: IAA content in leaf; Root.IAA: IAA content in root; Pn: Net photosynthetic rate; Tr: Transpiration rate; Gs: Stomatal conductance; Ci: Intercellular CO2 concentration; Leaf.PE: PE content in leaf; Root.PE: PE content in root; Leaf.BCF: Bioconcentration factor of PE in leaf; Root.BCF: Bioconcentration factor of PE in root; TF: Translocation factor; SOD: Superoxide dismutase; CAT: Catalase; POD: Peroxidase; MDA: Malondialdehyde content. [file Image1.jpeg]
